# Kinetically controlled shock-wave route to turbostratic boron-carbon-nitride nanosheets

**DOI:** 10.1016/j.isci.2026.116978

**Published:** 2026-07-30

**Authors:** Prakash Velampatti Selvaraj, Vijayanand Chandrasekaran

**Affiliations:** 1 Department of Chemistry, School of Advanced Sciences, Vellore Institute of Technology, Vellore, Tamil Nadu 632 014, India; 2 Department of Analytics, School of Computer Science and Engineering, Vellore Institute of Technology, Vellore, Tamil Nadu 632 014, India

**Keywords:** shock-wave, turbostratic nanostructure, kinetic trapping, non-equilibrium synthesis, solvent-free, catalyst-free, energy storage materials

## Abstract

Turbostratic boron carbon nitride (tBCN) is highly attractive for energy storage and catalysis owing to its rotationally disordered stacking and expanded interlayer spacing, yet direct synthesis of tBCN with high in-plane crystallinity remains challenging. Here, we demonstrate that a shock tube, as a non-equilibrium reactor, converts melamine and boric acid into tBCN nanosheets within 2–3 ms at reflected-shock temperatures > 6,000 K, with ultrafast quenching at ∼10^6^ K/s that kinetically traps a metastable turbostratic phase. The resulting nanosheets exhibit a few-layer morphology, expanded interlayer spacing, clear Moiré patterns, and high in-plane crystallinity, as confirmed by high-resolution transmission electron microscopy (HRTEM). Electrochemical analysis reveals a high double-layer capacitance (C_dl_) of 878 mF cm^−2^, substantially exceeding that of conventional BCN-based electrodes, which is attributed to the enlarged interlayer spacing and abundant defect sites in the turbostratic framework. This solvent-free, catalyst-free, single-step process offers a scalable and sustainable route to turbostratic nanomaterials for energy storage and optoelectronic applications.

## Highlights

• Shock waves offer a scalable, solvent- and catalyst-free route to tBCN nanosheets
• Shock waves rely on convective and radiative heating, independent of sample conductivity
• Millisecond shocks reach ∼6,000 K, quenching at ∼10^6^ K/s to trap metastable tBCN
• Electron microscopy reveals an enlarged 0.37 nm interlayer spacing and Moiré patterns

## Graphical abstract

**Figure:**
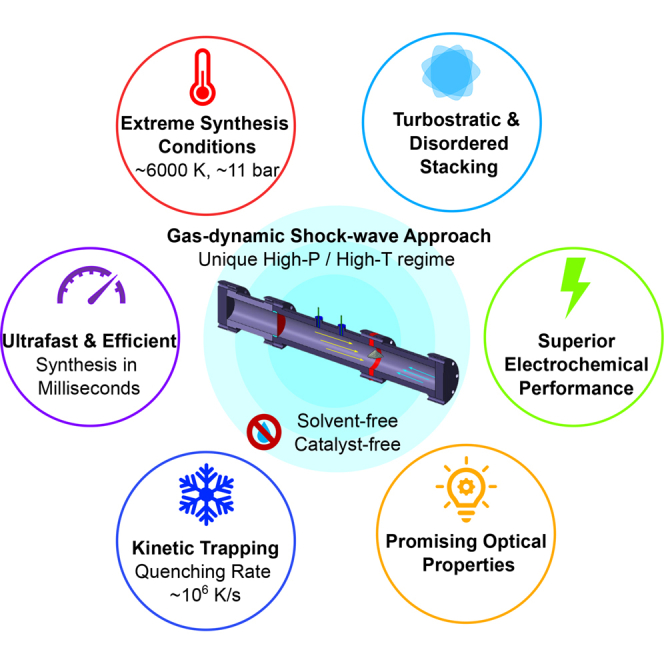

## Teaser

Materials science; Materials synthesis; Nanomaterials

## Introduction

Two-dimensional (2D) materials span a remarkable range of electronic properties, with graphene and hexagonal boron nitride (h-BN) representing the extremes of electrical conductivity and insulating behavior, respectively.^1^^,^^2^^,^^3^ Between these extremes, ternary boron-carbon-nitride (BCN) alloys bridge this gap by adjusting their composition.^3^^,^^4^ BCN can be tuned into semiconducting regimes while retaining high thermal stability and oxidation resistance.^5^ In ultrathin nanosheet form, BCN has attracted interest for supercapacitors,^6^^,^^7^ lithium-ion batteries,^3^^,^^8^ and electrocatalytic systems,^4^^,^^5^^,^^9^ where a combination of redox-active sites and composition-controlled band gaps can be tuned to enhance device performance.

Despite this potential, synthesizing high-quality BCN remains chemically and structurally challenging. Thermodynamically, the system favors B–N and C–C bonds over B–C and C–N bonds; so, prolonged high-temperature treatments and slow cooling naturally drive phase segregation into graphene-like carbon regions and h-BN-rich domains, rather than a homogeneous atomic alloy.^4^ Furthermore, canonical layered materials also tend to relax into thermodynamically favored stacking sequences (such as AA’ or AB), governed by interlayer van der Waals interactions, making it challenging to access turbostratic structures with rotational misalignment and enlarged interlayer spacing.^2^^,^^3^^,^^10^ Turbostratic BCN (tBCN) is particularly attractive because the lack of vertical alignment decouples neighboring layers, preserving the intrinsic band structure of individual sheets and facilitating exfoliation.^1^^,^^10^ However, BCN-based electrodes fabricated by conventional hydrothermal,^11^^,^^12^^,^^13^ chemical vapor deposition (CVD),^14^ and laser-scribing^15^ routes typically exhibit areal capacitances in the range of 40–180 mF cm^−2^, limited by small interlayer spacing and low active-site densities arising from equilibrium stacking. Even state-of-the-art multi-step composites, such as 3D BCN/reduced graphene oxide (rGO) broccoli structures^12^ and BCN nanotubes on carbon fibers,^16^ reach only 179 mF cm^−2^ and 177.1 mF cm^−2^, respectively. Achieving competitive electrochemical performance from a single-step, green synthesis route, therefore, remains an important open challenge.

Traditional synthesis strategies like CVD^17^ and static furnace pyrolysis^18^ demand high temperatures, long reaction times (hours to days), slow cooling, and often expensive substrates.^6^^,^^7^^,^^8^ Solvothermal methods, meanwhile, rely on hazardous solvents, large volumes, and complex multistep purification.^6^^,^^7^^,^^8^^,^^19^^,^^20^ The detailed experimental conditions, precursor choices, and specific limitations of these methods are summarized in Table 1. Under these near-equilibrium conditions, cooling rates are usually below ∼10 K/s, and the activation barriers for layer rearrangement can be easily overcome; so, the lattice relaxes into ordered, low-energy stacking modes, and the metastable turbostratic phase is not preserved.^21^ Although some low-temperature CVD approaches using single-source precursors suppress gross phase segregation,^17^ producing bulk quantities of highly crystalline tBCN remains challenging because this phase is intrinsically metastable and tends to revert to more ordered configurations unless its formation is kinetically trapped.

**Table 1:** Summary of conventional BCN synthesis routes reported in the literature, and their key limitations compared with the present shock-wave synthesis of tBCN nanosheets

Method	Precursors	Synthesis conditions	Reaction time	Product morphology	Notable properties/applications	Reference
Solvothermal	boric acid, 2,4,6-tri(2-pyridyl)-1,3,5-triazine (TPTZ), ethylene glycol	hydrothermal reactor (250 °C, 24 h); then N_2_ pyrolysis (400 °C/2 h); HCl wash	26 hrs	thin BCN sheets; irregular flaky morphology; small blisters on surface	specific capacitance: 343.1 F/g at 0.5 A/g; cycle stability: 90% after 3,000 cycles	Chen et al.^20^
Thermochemical synthesis (autoclave)	boric acid, acetone, ammonia solution	stainless-steel autoclave (700 °C, 24 h); natural cooling; ethanol wash	24 hrs	porous flower-like honeycomb BCN nanosheets; multilayer; irregular shape	optical stability (30 days); sharp PL emission at 3.64 eV; HER active; CV retention >90% after 5,000 cycles	Kaur et al.^19^
CVD (test-tube)	melamine, boric acid (1:0, 1:1, and 1:2 M ratios)	Muffle furnace; N_2_ flow; 300 °C (30 min) to 550 °C (4 h)	∼5 hrs total	transparent thin films; homogeneous; roughness <10 nm	tunable band gap; PL from near-UV (386 nm) to cyan (481 nm); applicable in photonics and solar cells	Giusto et al.^17^
CVD (planar)	melamine, boric acid (1:1 and 1:2 M ratios)	subtractive-reactive CVD; precursor at 500 °C, substrate at 550 °C; N_2_ flow	∼5 hrs total	transparent sp^2^ thin films	refractive index ∼2.2–2.28; UV-stable protective coatings	Giusto et al.^22^
Pulsed laser deposition (PLD)	B_4_C target, N_2_ gas	Si (100)/quartz substrate at 500°C; ND: YAG 355 nm laser; N_2_ atm	∼4 hrs	amorphous BCN thin films; thickness 875–1500 nm	deposition rate: 3.4–6.25 nm/min; N content saturates at ∼26 atom % (10 Pa); optical device applications	Yang et al.^23^
Hard templating	sucrose, borane-ammonia complex BAC, SBA-15 as hard template	chemical polymerization within SBA-15 nanochannels; carbonization (N_2_ atmosphere); HF washing to remove template	Hours-days	rod-shaped ordered mesoporous BCN (MBCN)	high surface area; specific capacitance: 296 F/g; CO_2_ capture: 27.14 mmol/g	Sathish et al.^24^
Dual ion beam sputtering (DIBS)	B_4_C target, Ar + N_2_ beam	DIBS chamber; Ar^+^ main beam; Ar/N_2;_ 40°C–60 °C	1.5–2 hrs	amorphous BCN thin films; thickness ∼300 nm; smooth surface	hardness up to 28 GPa; reduced elastic modulus up to 229 GPa; highly tunable hard coatings	Zhou et al.^25^
Facile precursor pyrolysis	trichloroborazine, methylaniline (1:1 M ratio)	sintering furnace 500 °C (30 min) to 1,000 °C (1 h); 1.5 MPa in N_2_	∼2–3 hrs	BCN nanoflakes (20 μm lateral, ∼10 nm thick); rippled/entangled morphology	oxidation onset at 810 °C; bulk-quantity; supercapacitor electrode potential	Zhang et al.^18^
Mechanically induced self-sustaining reaction (MSR)	B_2_O_3_, Mg, melamine	high-energy ball milling (600 rpm), self-propagating combustion, HCl wash	∼40 min	few-layer BCN nanosheets; crumpled/folded 2D morphology	rapid solid-state route; thermally stable	Jalaly et al.^26^
RF-ICP plasma	BH_3_NH_3_ (ammonia borane), CH_4_ gas, N_2_ gas	RF-ICP torch (15 kW, 62 kPa); Ar plasma carrier	seconds	2D BCN nanosheets (100–200 nm, 3–10 layers)	one-step process; band gap continuously tunable; applications in fuel cells, Li-ion batteries, supercapacitors	Alrebh and Meunier^27^
Flash Joule heating (FJH)	BH_3_NH_3_, carbon black	capacitor discharge (150 V); Ar atmosphere (1 atm); rapid cooling	100–600 ms	turbostratic BCN (f-BCN) 2D nanosheets	turbostratic structure aids mechanical exfoliation; high Cu corrosion protection for Cu (97% efficiency); 10^3^–10^4^ K/s cooling rate	Chen et al.^10^
Shock-wave processing	melamine, boric acid (1:2 M ratio)	reflected shock wave (∼6,000 K, 10.90 bar); rapid heating followed by rapid quenching	2–3 ms	turbostratic BCN nanosheets; few-layers; expanded interlayer; Moiré patterns	single-step, catalyst/solvent-free; rapid quenching rate: ∼10^6^ K/s; access to metastable turbostratic structures, unique high-P/high-T regime not accessible by plasma/FJH; C_dl_: 878 mF cm^−2^	this work

Accessing such metastable architectures, therefore, requires moving from a thermodynamically controlled regime to a kinetically controlled one. Rapid heating and quenching freeze the atomic structure in a metastable, mixed atomic arrangement before diffusion can restore it to the equilibrium.^10^ Ultra-fast, non-equilibrium methods, such as flash Joule heating (FJH) and laser-driven shock methods, have shown that temperature pulses on millisecond-to-microsecond timescales can stabilize disordered carbon-based and heteroatom-doped frameworks by outpacing long-range diffusion.^10^^,^^28^^,^^29^ Gas-dynamic shock tubes offer a complementary and comparatively unexplored route to similar conditions. They produce nearly step-like jumps in temperature and pressure with microsecond rise times, thereby creating a high-temperature environment that processes the sample homogeneously through convective and radiative heat transfer.^30^^,^^31^^,^^32^ Under these conditions, temperatures spike above 3,000 K and the pressure increases by tens of bars for a few milliseconds, providing the energy required to cleave strong bonds and drive rapid reorganization into non-equilibrium architectures.

Here, we demonstrate that tBCN nanosheets can be produced in a single-step shock-wave process from simple organic precursors, such as melamine and boric acid, by driving the reaction far from equilibrium under kinetically controlled conditions. The resulting material, characterized by advanced electron microscopy and spectroscopy, has an overall composition of BC_4_N_6.3_ and exhibits few-layer nanosheets with expanded interlayer spacing, Moiré patterns, and high in-plane crystallinity. The turbostratic structure enables these shock-synthesized nanosheets to achieve a high electrochemical double-layer capacitance (C_dl_) of 878 mF cm^−2^, substantially surpassing conventional BCN electrodes prepared using equilibrium methods and establishing a direct link between non-equilibrium synthesis kinetics and superior electrochemical performance. Shock-wave synthesis of tBCN, thus, opens new avenues for green synthesis of turbostratic stacks and for exploiting these materials for various energy applications.

## Results and discussion

### Shock-wave synthesis of tBCN nanosheets and formation mechanism

**Figure 1:**
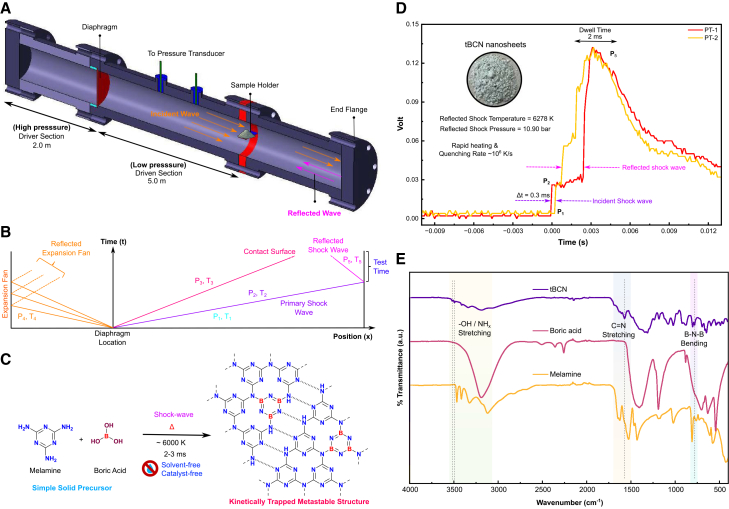
Shock-tube setup, precursor chemistry, and formation mechanism of tBCN nanosheets (A) Schematic diagram of the shock tube. (B) A typical x-t diagram illustrating the propagation of the shock wave along the tube. (C) Precursor mixture of melamine and boric acid (1:2 M ratio) and the corresponding structure of the motif derived by XPS. (D) Representative shock arrival times recorded by an oscilloscope; the inset shows the shock-synthesized sample. (E) Infrared spectra of the precursors and the shock-processed product.

The synthesis of tBCN is driven by the complex interplay of incident and reflected shock waves, which generates a dual-stage thermal profile that efficiently vaporizes and recondenses the melamine and boric acid (1:2) precursor mixture into the BCN framework. Rapid pressurization of helium in the driver section forces the diaphragm to rupture (Figure 1A), triggering an incident shock wave. As the shock wave travels with a velocity of ∼1.68 km/s, it rapidly compresses and heats the driven (Ar) gas to 2,813 K (T_2_). The incident shock wave may initiate the precursor to sublimate. Once the incident shock wave strikes the end wall, a reflected shock wave forms (Figure 1B). This reflected shock wave moves toward the driver section, heating the driven gas again and raising its temperature to 6,278 K (T_5_), and the pressure to 10.90 bar (P_5_). The flow is stationary behind the reflected shock wave, and the high temperature T_5_ and pressure P_5_ are maintained for 2–3 ms. The hot Ar gas transfers its energy to the sample through convection and radiation. Under these extreme transient conditions, both melamine and boric acid decompose, stripping the –OH groups from boric acid and –NH_2_ groups from melamine, and releasing volatile byproducts (H_2_O and NH_3_) that are carried away by the gas flow. As the expansion wave propagates, the system undergoes an ultrafast quench with cooling rates of ≥10^6^ K/s. This rapid thermal arrest inhibits the thermodynamic segregation of B, C, and N atoms, thereby forcing them to nucleate into a chemically intermixed, kinetically trapped B–C–N nanosheet framework (Figure 1C). The subsequent cooling phase determines the nanosheet’s final morphology. A rapid thermal process in a shock tube mimics flash-heating techniques but relies on gas-dynamic heating, rather than electrical discharge, thereby enabling solvent-free, catalyst-free processing of solid precursors.^10^

This gas-dynamic evolution of the synthesis cycle is depicted in the position-time (x-t) diagram (Figure 1B). To verify these regimes, shock propagation was observed in real time, using piezoelectric pressure transducers mounted in the driven section. The pressure traces indicating the dynamic pressure history are measured with an oscilloscope, as shown in Figure 1D, and used to calculate the incident shock velocity ν_s_. Conditions across the shock front were evaluated using the Rankine-Hugoniot jump relations^33^ (Equations 2, 3, and 4). Using these standard normal-shock relations, we calculated the reflected-shock wave parameters, including the stagnation temperature (T_5_) and pressure (P_5_). The results are summarized in Table 3.

### Structural and spectroscopic characterization

Fourier transform infrared (FTIR) spectra provide a clear picture of the chemical transformation from the precursor to the shock-synthesized BCN product. As shown in Figure 1E, the precursors exhibit a broad absorption band between 2,700 and 3,700 cm^−1^, characteristic of –OH and –NH_2_ stretching modes. Following shock exposure, this feature is significantly suppressed, indicating the effective removal of these functional groups. However, refined spectral analysis reveals sharp, discrete resonances at 3,518 and 3490 cm^−1^, which are attributed to a residual supramolecular hydrogen-bonded network, likely stabilizing the edges of the newly formed domains.^17^ In the fingerprint region, the development of the hexagonal lattice is evidenced by strong peaks at 1,380 and 780 cm^−1^, corresponding to the in-plane B–N stretching and out-of-plane B–N–B bending modes, respectively.^4^ Crucially, the spectra also exhibit additional bands in the 1,000–1,250 cm^−1^ range, along with shoulders near 1,600 cm^−1^, which are attributed to B–C and C=N stretching modes, consistent with the formation of a chemically bonded sp^2^ B–C–N framework, rather than a physical mixture of BN and separate carbon phases.^5^^,^^17^

**Figure 2:**
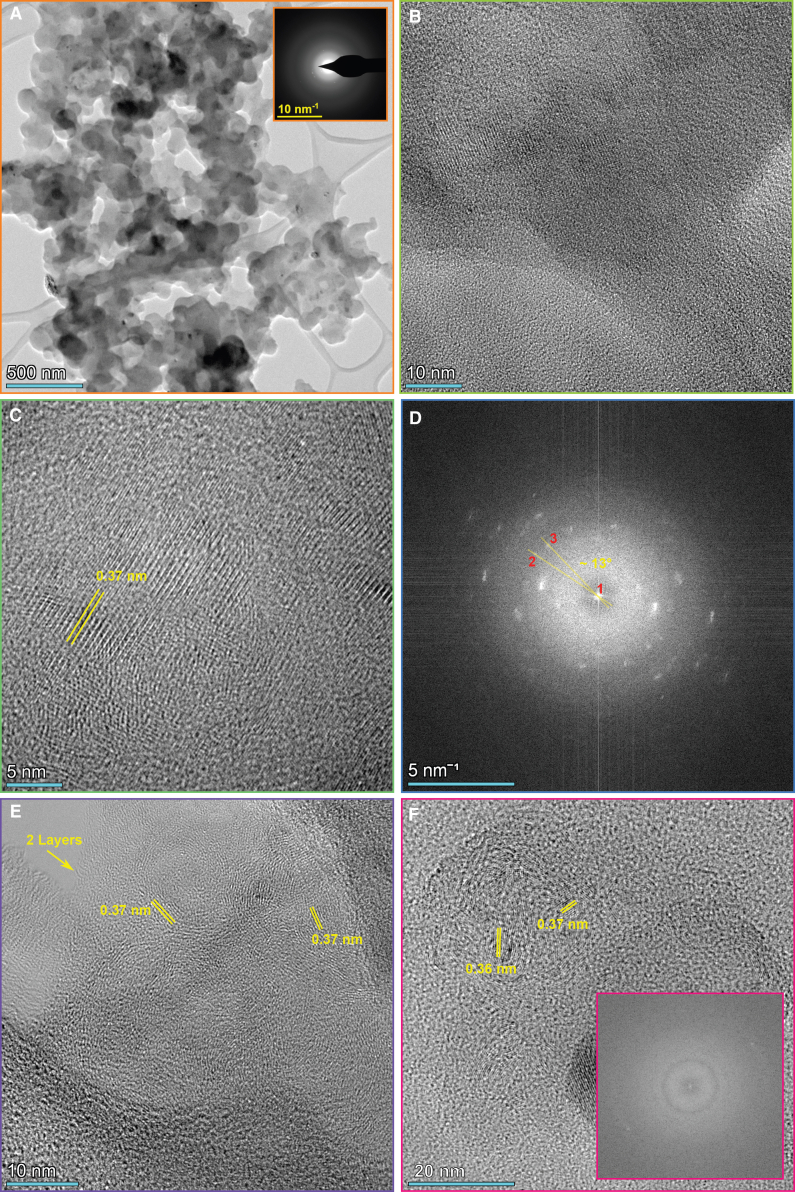
TEM and HRTEM analyses of the shock-synthesized tBCN nanosheets (A) TEM image of tBCN nanosheets (scale bar: 500 nm); the inset shows the SAED pattern indicative of a layered, turbostratic structure (scale bar: 10 nm^−1^). (B) HRTEM image highlighting locally ordered lattice fringes and high in-plane crystallinity within the turbostratic framework (scale bar: 10 nm). (C) Magnified region of a tBCN nanosheet with well-resolved fringes (scale bar: 5 nm). (D) Corresponding FFT pattern indicating a ∼13° tilt between stacked planes, consistent with turbostratic stacking (scale bar: 5 nm^−1^). (E) HRTEM image revealing that the shock-synthesized nanosheets consist of only two to a few atomic layers (scale bar: 10 nm). (F) TEM image highlighting rotation-induced in-plane Moiré patterns within the turbostratic framework (scale bar: 20 nm); the inset shows the corresponding FFT pattern.

Transmission electron microscopy (TEM) reveals the morphological evolution of the shock-processed material, showing large-scale, electron-transparent nanosheets (Figure 2A). The corresponding selected-area electron diffraction (SAED) pattern shows diffuse rings with distinct spots (inset, Figure 2A), indicating rotational disorder between stacked layers, typical of turbostratic architectures.^1^ High-resolution TEM (HRTEM) imaging (Figures 2B and 2C; Figures S1B–S1D) further resolves this microstructure, revealing continuous, well-defined lattice fringes and high in-plane crystallinity within the turbostratic framework. The fast Fourier transform (FFT) of a selected area (Figure 2D) shows a distinct spot, with an angular offset of 13°, confirming that the neighboring layers are rotationally misaligned and that the stacking is turbostratic,^1^^,^^10^^,^^17^ clearly distinguishing the material from the rigid Bernal stacking of bulk h-BN. HRTEM imaging of the sheets exhibits largely electron-transparent regions and exhibits folded edges, consistent with a two- to a few-layer thickness (Figure 2E), providing further evidence for layered stacking and interlayer expansion. Line profile analysis across these fringes (Figures 2C, 2E, and 2F) yields an expanded average interlayer spacing of ∼0.37 nm. This value is significantly larger than the 0.34 nm characteristic of stoichiometric h-BN.^10^^,^^27^ This lattice expansion is attributed to heteroatom incorporation and local bond-length mismatch (e.g., C–N vs. B–N), which weakens van der Waals interactions between the adjacent sheets and prevents tight stacking.

Furthermore, HRTEM imaging (Figure 2F) reveals complex Moiré interference fringes spanning the nanosheet surface, indicative of the absence of Bernal (AB) stacking and the presence of rotational stacking disorder, with random rotational angles between adjacent layers.^1^^,^^10^ The corresponding FFT pattern is shown in the inset of Figure 2F. Such turbostratic structures with expanded interlayer spacing are advantageous for electrochemical applications, as they facilitate ion transport and provide abundant defect sites.^10^ tBCN can possess an arbitrary number of stacked layers, whose optical and electronic states remain effectively decoupled.^21^ This leads to electronic behavior that more closely mirrors that of monolayer BCN than that of conventional multilayer BCN, due to reduced interlayer coupling.^1^^,^^10^ Finally, elemental analysis by energy-dispersive X-ray spectroscopy (EDS; Figure S1E) of the shock-processed samples confirmed the presence of the ternary B–C–N phase, with B, C, and N as the primary elements.

**Figure 3:**
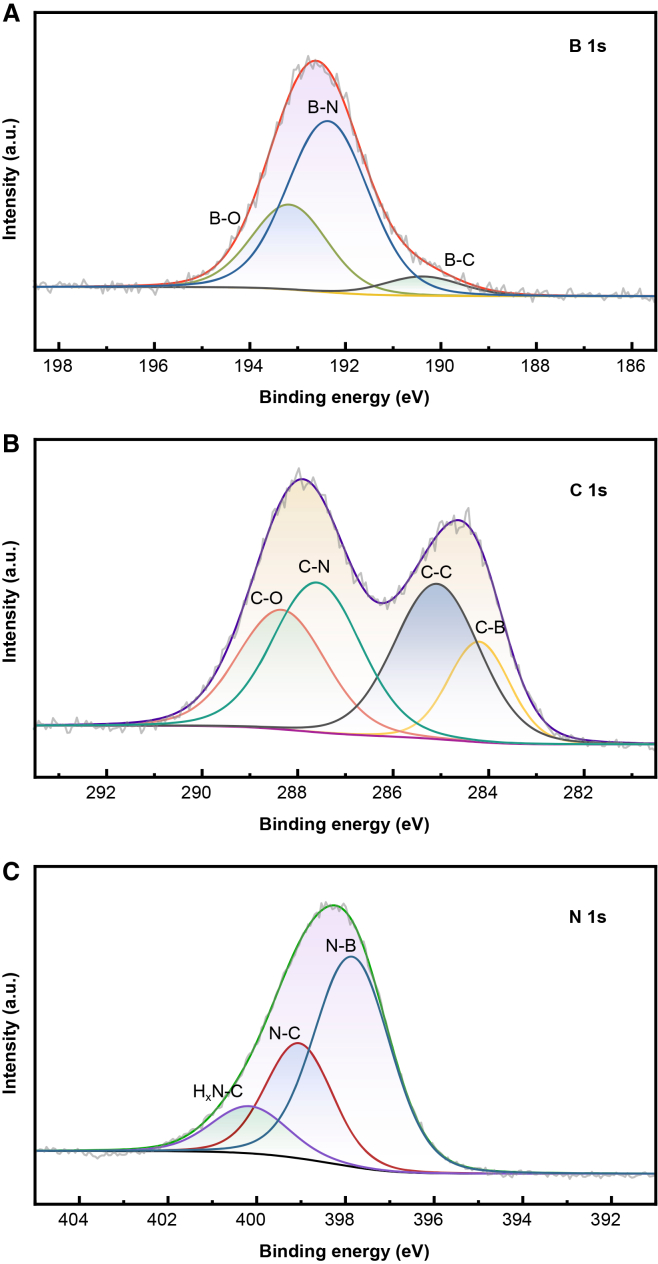
XPS core-level spectra of shock-synthesized tBCN nanosheets (A) B 1s core-level spectrum resolved into signals associated with B–N, B–C, and B–O coordination environments. (B) C 1s spectrum displaying contributions from C–B, C–C, C–N, and C–O species, indicating carbon incorporation into the B–C–N framework. (C) N 1s spectrum comprising N–B, N–C, and H_x_N–C peaks, consistent with an intermixed, conjugated tBCN network.

To elucidate the atomic-level connectivity and elemental stoichiometry of the shock-synthesized tBCN nanosheets, we performed high-resolution X-ray photoelectron spectroscopy (XPS). The wide-scan spectrum in Figure S2A reveals boron, carbon, and nitrogen as the dominant elements, confirming the formation of a ternary B–C–N alloy. The observed oxygen contribution likely originates from surface oxidation or atmospheric adsorbates (Figure S2B). During shock processing, the precursor mixture undergoes sublimation, and most oxygen-containing species are evaporated as water vapor upon venting the system. However, a minor residual fraction of oxygen remains in the product. HRTEM-EDS analysis is consistent with this finding (Table S1), revealing oxygen as a minor constituent contributing ∼5% to the overall elemental composition. Deconvolution of the B 1s spectrum in Figure 3A reveals three distinct components, collectively ruling out simple phase segregation into discrete BN and carbon domains. The peak at 190.3 eV is assigned to N–B–C coordination, providing direct evidence of ternary alloying, while the component at 192.3 eV is attributed to HN−B−N_2_/sp^2^ B−N bonding environments, indicating extensive integration of boron into nitrogen-rich domains.^17^^,^^34^ An additional component at 193.1 eV corresponds to B–O bonding, likely arising from surface oxidation at defect sites within the turbostratic framework.^17^^,^^34^ The C 1s spectrum (Figure 3B) is dominated by a peak at 287.6 eV assigned to N–C=N/C–NH environments,^17^^,^^22^ consistent with triazine- or carbon nitride-like units, and a peak at ∼285.0 eV attributed to sp^2^ C–C bonding, including adventitious carbon.^34^ Additional contributions at 288.3 and 284.2 eV are assigned to C–O and B–C bonding, respectively,^22^^,^^34^ reflecting oxidative surface bonding at the defect sites and direct boron–carbon coordination within the B–C–N lattice. The N 1s spectrum (Figure 3C) displays two dominant peaks at 397.8 and 399.0 eV, assigned to N–B and C–N=C environments, respectively.^17^^,^^22^^,^^34^ A lower-intensity peak at 400.1 eV is attributed to terminal C–NH–C groups.^17^ The co-existence of these three nitrogen environments confirms that nitrogen bridges both boron- and carbon-centered sites within a conjugated lattice, an intermixed B–C–N network.

Quantitative analysis of the XPS spectra yields an overall composition close to BC_4_N_6.3_ (B/C/N ratio: 8.75:35.66:55.59), in excellent agreement with values reported previously for low-temperature CVD-grown BCN thin films.^17^ Based on this stoichiometry, we propose that a structural motif can be interpreted as a BN unit surrounded by four C_3_N_4_ units.^17^

$$3\;\left({\text{BC}}_{4}N_{6.3}\right)\approx B_{3}C_{12}N_{19}\equiv B_{3}N_{3}+4\;\left(C_{3}N_{4}\right)$$

The model structures shown in Figure 1C illustrate representative arrangements of triazine and borazine units, interconnected by hydrogen bonds, consistent with the bonding configurations inferred from the XPS and FTIR spectra.

### Optical properties

**Figure 4:**
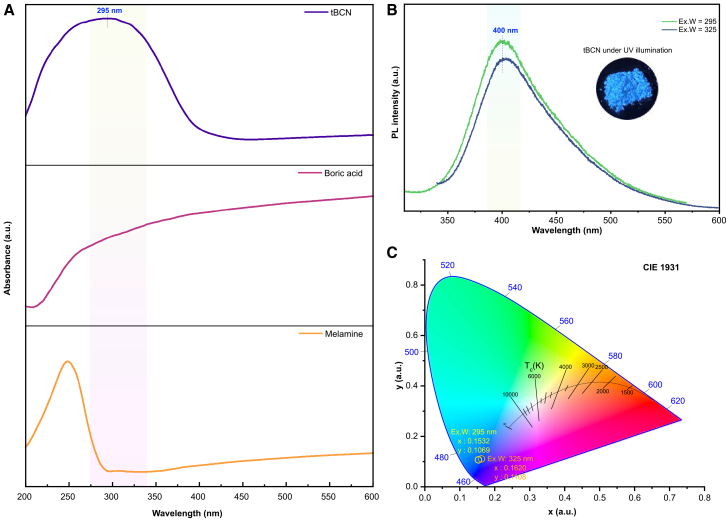
Optical properties of the shock-synthesized tBCN nanosheets (A) UV-vis absorption spectrum of the precursors and tBCN nanosheets. (B) Photoluminescence spectrum of tBCN nanosheets recorded at the excitation wavelengths of 295 and 325 nm; inset, a photograph of the tBCN under UV illumination showing visible blue emission. (C) Corresponding emission color of the nanosheets plotted on the CIE 1931 chromaticity diagram.

To elucidate how shock-induced B–C–N alloying affects the electronic structure, we characterized the material, using diffuse reflectance UV-visible spectroscopy (Figure 4A). The spectra showed a distinct absorption edge at 295 nm, with a tail extending into the visible range, typical of a semiconducting material with defect- or alloy-derived states within the band gap. Tauc plot analysis yielded an optical band gap of 3.15 eV (Figure S3), intermediate between graphene (0 eV) and h-BN (∼5.5–6.0 eV), and consistent with compositionally mixed BCN alloys.^17^ The significant decrease in the band gap relative to pristine h-BN is attributed to the incorporation of C–N bonds.

We explored the radiative property by using photoluminescence (PL) spectroscopy. Under excitations at 295 and 325 nm, the nanosheets exhibited a robust, prominent emission peak centered at 400 nm (3.10 eV; Figure 4B).^35^ Interestingly, the optical band gap (3.15 eV) and this emission maximum were separated by a remarkably narrow Stokes shift of 0.05 eV. Such a remarkably small difference of 0.05 eV strongly implies that radiative recombination occurs primarily at localized states, likely arising from sp^2^ C–N and B–C–N defect motifs. The tBCN nanosheets under UV illumination exhibited intense deep-blue emission (inset of Figure 4B), underscoring the unique advantage of this non-equilibrium shock process: it kinetically traps these optically active heteroatom substitutions, generating structural features that are entirely absent in both the deep-UV luminescent h-BN and the non-luminescent graphene.

We mapped the chromaticity coordinates onto the CIE 1931 color space,^17^ as shown in Figure 4C, to rigorously quantify the emission’s purity and color profile. When excited at 295 nm, the tBCN nanosheets exhibited high-purity deep-blue emission (with coordinates of 0.1532 and 0.1069). Shifting the excitation wavelength to 325 nm produced only a marginal shift to (0.1620, 0.1108). The 400 nm peak remained stationary alongside this minimal coordinate shift, strongly indicating a highly homogeneous distribution of radiative centers throughout the turbostratic layers. It is very interesting to compare the position of the measured coordinates with the NSTC (National Television System Committee) blue primary standard (0.14, 0.08).^36^ The measured values (0.1532, 0.1069) are very close to the NSTC blue primary standard. Such close proximity highlights the exceptional color saturation unlocked by the shock-wave synthesis. Furthermore, the *y* coordinate serves as a sensitive proxy for spectral contamination by lower-energy transitions, such as green or yellow. While many carbon-based nanomaterials suffer from *y* values > 0.20 due to surface oxidation or mid-gap states,^37^ our tBCN suppresses this contamination, maintaining a stringent *y* value of 0.1108. This spectral cleanliness meets the stringent requirements of a high-performance blue phosphor for next-generation displays and optoelectronics. We attribute this high color purity to the unique synthesis kinetics of the shock wave route. The rapid, non-equilibrium nature of this process appears to bypass the formation of non-radiative recombination centers or secondary oxidative phases typically found in slower, multistep conventional syntheses.^6^

### Electrochemical performance

**Figure 5:**
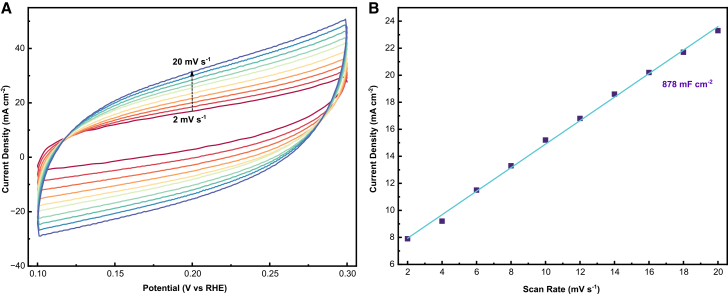
Estimation of electrochemical C_dl_ (A) Cyclic voltammograms acquired in a non-Faradaic potential window (0.10–0.30 V vs. RHE) at scan rates ranging from 2 to 20 mV s^−1^ (in 2 mV s^−1^ increments). (B) The corresponding linear plot of ΔJ/2 versus scan rate was used to determine C_dl_.

The turbostratic architecture of the shock-synthesized tBCN nanosheets results in superior electrochemical performance. Details of the electrochemical measurements are provided in STAR Methods. To assess the electrochemically active surface area (ESCA) of the as-prepared tBCN nanosheets, C_dl_ was used as a proxy. Cyclic voltammetry (CV) was conducted within a non-Faradaic potential window at incrementally increasing scan rates from 2 to 20 mV s^−1^ (in 2 mV s^−1^ increments) in the potential range of 0.10–0.30 V vs. the reversible hydrogen electrode (RHE) (Figure 5A). The C_dl_ was extracted from the slope of the linear plot of capacitive current density (j) versus scan rate (*ν*), according to the following equation:

(Equation 1) $$j=C_{dl}\cdot \nu$$

The average of the anodic and cathodic current densities at 0.20 V vs. RHE was plotted as a function of scan rate, from which C_dl_ was derived (Figure 5B). The tBCN electrode exhibited a notably high C_dl_ of 878 mF cm^−2^, substantially exceeding that of previously reported BCN-based electrocatalysts and indicating a significantly enlarged ESCA available for catalytic reactions. This exceptional capacitance can be attributed to the synergistic effect of the turbostratic architecture. The enlarged interlayer spacing relative to conventionally stacked h-BN facilitates rapid ion transport and intercalation, while the abundant rotational defect sites and high in-plane crystallinity, kinetically trapped by the ultrafast cooling, provide a high density of ESCAs.

The electrochemical performance of tBCN is benchmarked against BCN-family electrode materials in Table 2. The tBCN electrode (878 mF cm^−2^) delivered markedly superior performance to that of all multi-step composites prepared by conventional equilibrium routes (41–179 mF cm^−2^)^11^^,^^12^^,^^13^^,^^14^^,^^15^ and is competitive with ACC@BCN (1,018 mF cm^−2^),^38^ despite the latter requiring multi-step calcination at 900 °C from an activated carbon fiber cloth substrate. This comparison demonstrates that the non-equilibrium synthesis kinetics of the shock-wave route, rather than compositional additives or substrate engineering, are the primary driver of enhanced electrochemical performance, achieved through kinetic trapping of a highly active turbostratic microstructure.

**Table 2:** Typical experimental shockwave parameters obtained from Rankine-Hugoniot jump relations

Precursor ratio	Driver gas (He) pressure in bar	Driven gas (Ar) pressure in bar	Velocity (km/s)	M	T(K)		P_r_ (bar)
					T_i_	T_r_	
Melamine + boric acid (1:2)	15.2	0.06	1.68	5.23	2813	6278	10.90

M, incident Mach number; T_i_ and T_r_, incident and reflected shock temperatures, respectively; P_r_, reflected shock pressure.

### Green chemistry and sustainability

Synthesizing tBCN nanosheets with high in-plane crystallinity presents a fundamental challenge, as conventional processing routes and precursor choices typically drive the system toward phase segregation or structural disorder. A detailed comparison of reaction kinetics, energy efficiency, and environmental impact between the shock-wave route and established methods is provided in Table 1 and Methods S1.

Conventional gas-phase techniques often rely on highly reactive precursors (e.g., BCl_3_/NH_3_ or ammonia borane) that react rapidly to form BCN. However, the resulting local supersaturation and uncontrolled nucleation frequently yield amorphous products with high defect densities. Furthermore, furnace-based CVD and pyrolysis operate near thermodynamic equilibrium (900°C–1100 °C, hours-long reaction times),^18^^,^^22^ where atomic diffusion rates are sufficient to relax the system toward its global minimum-energy phase-segregated graphene and h-BN.

In contrast, our shock-wave technique operates in a kinetically controlled regime. Catastrophic, rapid heating drives the chemical reaction of the precursor, during which extensive B–C–N mixing occurs, followed by rapid cooling. Both heating and cooling rates are ≥10^6^ K/S, trapping atoms in their mixed configuration before phase segregation can proceed. This pathway accesses metastable regions of the BCN phase space that are difficult to reach with equilibrium methods.

Previous work on tBCN synthesized via FJH has demonstrated a highly efficient (3.1 kJ/g) process that occurs in less than a second, but it relies on resistive heating, which requires electrically conductive precursors.^10^ Consequently, insulating precursors such as ammonia borane require conductive additives (e.g., carbon black), which serve as both a carbon source and a current carrier.^10^ This complicates the compositional control and purity and necessitates a secondary high-temperature calcination step (675 °C in air) to remove residual carbon. Our approach bypasses this constraint entirely. Because shock-wave processing relies on convective and radiative heating, the heating mechanism is independent of the sample’s electrical conductivity. This enables the direct processing of fully insulating powders (melamine and boric acid) without electrically conductive additives, offering an advantage for synthesizing dielectric or semiconducting BCN phases. Moreover, the shock tube technique achieves a unique combination of high temperature and high pressure that complements the existing plasma/FJH techniques.

This shock-wave synthesis is benchmarked against the twelve green chemistry principles (Methods S1) and the targeted UN sustainable development goals (SDGs), demonstrating a clear advantage over conventional synthesis routes.^43^^,^^44^

In direct alignment with SDG 12 (“responsible consumption and production”),^45^ this shock-wave methodology achieves high atom economy and minimizes waste generation. Conventional solvothermal routes consume liters of organic solvents, such as dimethylformamide (DMF) or ethanol, and inherently produce high E-factors and environmental costs.^6^^,^^8^ By operating a completely dry, solvent-free system, our approach entirely eliminates this chemical overhead. Utilizing abundant, low-toxicity industrial feedstocks (melamine and boric acid) avoids hazardous reductants and halide precursors, producing only benign gaseous byproducts (H_2_O or NH_3_) rather than corrosive HF or HCl.^46^

In support of SDG 6 (“clean water and sanitation”),^47^ this completely dry synthesis (water-free) method yields a cleaner value chain for advanced water remediation. The shock process forces the formation of a defect-rich turbostratic framework, providing numerous adsorption sites. When deployed in aqueous environments, these structures are known to aggressively capture heavy metals and organic pollutants, offering a high-performance, scalable tool to help curb industrial discharge and restore water quality.^48^

Addressing SDG 7 (“affordable and clean energy”),^49^ the ultrafast, millisecond-scale synthesis significantly reduces the energy footprint compared with prolonged furnace heating. Furthermore, the product, characterized by enlarged interlayer spacing and turbostratic disorder, enables rapid ion transport and intercalation.^10^ This enables BCN-based electrodes to achieve capacitances exceeding those of conventional carbon-based materials, thereby contributing to the development of low-cost, high-performance energy-storage systems essential for integrating renewable energy.

### Limitations of the study

Although the shock-wave synthesis route demonstrated here offers considerable advantages in reducing reaction time and improving sustainability, several limitations remain. First, the most significant constraint inherent to shock-tube experiments is the available reaction time at the peak pressure P_5_ and temperature T_5_. This time interval is defined as the period between the reflected shock’s arrival at the end wall and the subsequent arrival of the expansion wave, as illustrated in the x-t diagram (Figure 1B).

This reaction time can be increased by lengthening the driver section. Even so, the reaction time will be in milliseconds, not seconds. For reactions requiring long reaction times, the sample can be exposed to multiple shocks. However, this is a time-consuming process. Second, the reaction was carried out on a milligram scale (0.25 g sample loading). Gram- or kilogram-scale production has yet to be demonstrated. Third, the electrochemical characterization reported here is confined to C_dl_ measurements as a proxy for ECSA in a three-electrode configuration. Full device-level performance (e.g., symmetric supercapacitor cycling stability, rate capability, and energy and power density) remains to be evaluated. Addressing these limitations in future work is essential to fully realize the potential of shock-wave-synthesized tBCN nanosheets across energy storage, catalysis, and optoelectronic platforms.

## Resource availability

### Lead contact

Requests for further information, resources, and reagents should be directed to and will be fulfilled by the lead contact, Vijayanand Chandrasekaran (vijayanand.c@vit.ac.in).

### Materials availability

Materials used in the study are commercially available.

### Data and code availability

• All data reported in this paper will be shared by the lead contact upon reasonable request.
• No new code was generated during the course of this study.
• Any additional information required to reanalyze the data reported in this paper is available from the lead contact upon reasonable request.

## Acknowledgments

P.V.S. acknowledges support from the Council of Scientific and Industrial Research (10.13039/501100001412CSIR), India, through a Senior Research Fellowship (SRF-Direct, file no. 09/0844(18755)/2024-EMR-I). V.C. thanks the 10.13039/501100004728Vellore Institute of Technology (10.13039/100019904VIT) for a Seed Grant supporting this work. The authors acknowledge the Micro Nano Characterization Facility (MNCF) at the Indian Institute of Science, Bengaluru, for HRTEM measurements and the Central Instrumentation Facility (CIF) at IISER-Thiruvananthapuram for XPS characterization. The authors also thank Dr. Arun Prasad Murthy, Associate Professor, School of Advanced Sciences, VIT-Vellore, for his support with the electrochemical characterization.

## Author contributions

Conceptualization and methodology, P.V.S. and V.C.; formal analysis, data curation, and visualization, P.V.S.; validation and investigation, P.V.S. and V.C.; writing – original draft, P.V.S..; writing – review & editing, P.V.S. and V.C.; funding acquisition, project administration, resources, and supervision, V.C.

## Declaration of interests

The authors declare no competing interests.

## STAR★Methods

### Key resources table

**Table:** 

REAGENT or RESOURCE	SOURCE	IDENTIFIER
**Chemicals, peptides, and recombinant proteins**		

Melamine (>97.5%)	SD Fine-Chem Ltd.	CAS #: 108-78-1
Boric acid (>99.5%)	Sigma-Aldrich	CAS #: 10043-35-3
Nafion	Sigma-Aldrich	CAS #: 31175-20-9
Acetone	Sisco Research Laboratories Pvt. Ltd.	CAS #: 67-64-1
Helium gas (He, 99.999%)	Rana Industrial Gases and Products, Chennai	N/A
Argon gas (Ar, 99.999%)	Rana Industrial Gases and Products, Chennai	N/A
Mylar sheet (250 μm thick)	Purchased from Local Vendors	N/A

**Software and algorithms**		

Origin software	OriginLab Corporation	https://www.originlab.com
HRTEM image processing	ImageJ	https://imagej.net/ij
CHI electrochemical software	CH Instruments	https://www.chinstruments.com
Vector graphics editor	Inkscape	https://inkscape.org

### Experimental model and study participant details

There are no experimental models (animals, human participants, plants, microbe strains, cell lines, primary cell cultures) used in this study.

### Method details

#### Materials

Reagent-grade melamine (>97.5%, SD Fine-Chem) and boric acid (>99.5%, Sigma-Aldrich) were obtained from commercial suppliers and used as received. Ultra-high-purity helium (He, 99.999 %) and argon (Ar, 99.999 %) gases were supplied by Rana Industrial Gases and Products, Chennai, and used as the working and driver gases in the shock-tube experiments. Before each run, the inner walls of the shock tube were cleaned with analytical-grade acetone and dried to minimize contamination from previous shots. A 250 μm thick Mylar sheet was used as the diaphragm separating the high-pressure driver section from the low-pressure driven section.

#### Shock tube configuration

The shock tube has been conventionally used in combustion, gas-phase chemical kinetics, high-enthalpy aerodynamics, and astrochemistry research.^30^^,^^31^^,^^32^^,^^33^^,^^50^ Here, we use a stainless-steel shock tube to synthesize tBCN nanosheets (Figure 1A). The shock tube working principle, configuration, and operating procedures were described in our previous publication.^33^ Briefly, the setup comprises a two-meter-high pressure driver section and a five-meter driven section, separated by a Mylar diaphragm (2 sheets). The tube’s internal and external diameters are about 8 cm and 11.5 cm, respectively. A manually operated ball valve is positioned 5 m into the driven section. Just beyond this valve, a powder sample holder is coupled to a 0.6 m long reaction chamber closed with an end flange. The powder sample holder is a 2-mm-thick horizontal plate centered within the tube's cross-section. Melamine and boric acid (1:2 molar ratio, about 0.25g total) were mixed and spread on the sample holder to improve shock-wave interaction with the sample particles. Before each experiment, the driven section was pumped to 5 × 10^−2^ mbar at a slow rate to prevent sample disturbance. The driven section was then filled with argon to atmospheric pressure and pumped again. This process was repeated three times before the experiment. Finally, the driven section was filled with ultrahigh-purity argon to a pressure of 0.06 bar. He and Ar served as the driver and driven gases, respectively. The driver section was then rapidly pressurized with He gas until diaphragm rupture at approximately 15.2 bar, generating an incident shock wave in the driven section. Two piezoelectric pressure transducers (PCB Piezotronics; sensitivity ∼0.5 mV/PSI) were mounted 30 cm apart along the driven section and connected to a digital storage oscilloscope (Tektronix, TDS2014B) to determine the incident shock velocity. The primary shock wave interacts with the sample and the argon gas in the reaction chamber, heating the argon gas to pressure P_2_ and temperature T_2_. The primary shock wave subsequently reflected from the end flange. It then propagated back through the already-shocked gas, reheating the sample and the argon gas to pressure P_5_ and temperature T_5_. All key shock-wave parameters, including velocity, P_5_, and T_5_, were calculated from standard normal-shock relations, and the results are summarized in Table 3. After the experiment, the tube was held at high pressure for several hours to allow the particles to settle. After careful venting to atmospheric pressure, solid products were collected from the end and inner walls for *ex-situ* analysis. The resulting material is referred to as turbostratic Boron-Carbon-Nitride (tBCN). All spectroscopic, microscopic, and electrochemical characterizations of the tBCN nanosheets were performed without any post-purification.

**Table 3:** Comparison of electrochemical performance with recently reported electrode materials

Electrode material	Precursors used	Fabrication methods	Electrolyte	Areal capacitance (mF cm^−2^)	Reference
NiCo-OH/ZnO-nanorod/CNT-yarn	CNT yarn, ZnO precursors, Ni/Co precursors	two-step hydrothermal growth	PVA-LiOH gel/1 M LiOH	1,065 (at 5 mV s^−1^)	Le et al.^39^
ACC@BCN	activated carbon fiber cloth (ACC), urea, boric acid, P123	impregnation + calcination (900 °C)	PVA-KOH gel/6 M KOH	1,018 (at 1 mA cm^−2^)	Shi et al.^38^
Carbon nanofibers yarn (CNY)@ polypyrrole (PPy) @ rGO	electrospun polyacrylonitrile (PAN), pyrrole, GO	carbonization, electrochemical deposition, reduction	PVA/H_3_PO_4_ gel	836.87 (at 2 mV s^−1^)	Chen et al.^40^
Graphene fibers (GF90)	graphite foil, ammonium persulfate	electrochemical exfoliation	PVA/H_3_PO_4_ gel/1 M H_2_SO_4_	247.6 (at 2 mA cm^−2^)	He et al.^41^
3DBCN/rGO broccoli	graphite powder, melamine, boric acid, sucrose	hydrothermal, calcination, laser engraving	PVA-H_2_SO_4_ gel	179 (laser engraved)	Tu et al.^12^
BCNNTs	carbon fibers, urea, boric acid	*in situ* calcination/pyrolysis	EMIM·BF_4_/PVDF-HFP gel	177.1 (at 5 mA cm^−2^)	Liang et al.^16^
BNCNT-CC	carbon cloth, ferrocene, ammonia borane	spray pyrolysis thermal CVD	PVA/H_2_SO_4_ gel	106.8 (at 1 mA cm^−2^)	Paul et al.^14^
MXene/BCN microflowers	melamine, boric acid, sucrose, LiF, Ti_3_AlC_2_, HCl	etching, hydrothermal, annealing, screen printing	PVA-H_2_SO_4_ gel	89 (at 0.5 mA cm^−2^)	Tu et al.^11^
BCNN (nanomesh)	milk powder (protein), boric acid	sol-gel, carbonization (900 °C), laser cutting	PVA/H_3_PO_4_ gel/EMIMBF_4_/PVDF-HFP ionogel	80.1 (at 0.25 mA cm^−2^)	Zhang et al.^15^
Laser-patterned BCN	boric acid, glucose, cyanamide	thermal annealing + CO_2_ laser scribing	PVA-H_2_SO_4_ gel	72 (at 0.15 mA cm^−2^)	Karbhal et al.^42^
3D-BCN microspheres	sucrose, boric acid, melamine	hydrothermal, annealing, screen printing	PVA-KOH gel	41.6 (at 0.05 mA cm^−2^)	Tu et al.^13^
tBCN nanosheets	melamine, boric acid	single-step shock-wave synthesis	1M KOH	878 (at 10 mV s^−1^)	this work

#### Shock-wave parameters calculation

Piezoelectric pressure transducers, located at fixed intervals L (30 cm) in the driven section of the shock tube, recorded the shock arrival times as shown in Figure 1D. The velocity was determined from the transit time (Δt) between the two pressure transducers, corresponding to the shock front travelling the distance (Δx) between the pressure sensors, according to ν_s_= Δx/Δt. The corresponding incident Mach number, M, was then obtained by normalizing this velocity with respect to the pre-shock sound speed (c_1_) in the driven gas:

(Equation 2) $$M=\frac{v_{s}}{c_{1}}=\frac{v_{s}}{\sqrt{\gamma {\text{RT}}_{1}}}$$

Where *γ* is the adiabatic index, R is the specific gas constant, and T_1_ is the initial temperature of the test gas.

#### Reflected shock-wave conditions

Using the calculated incident Mach number, M, as input to the Rankine-Hugoniot jump relations,^33^ the reflected-shock temperature T_5_ and pressure P_5_ were obtained from the following equations.

(Equation 3) $$\frac{T_{5}}{T_{1}}=\frac{T_{5}}{T_{2}}\times \frac{T_{2}}{T_{1}}=\left(\frac{p_{5}}{p_{2}}\right)\left(\frac{\frac{\gamma +1}{\gamma -1}+\frac{p_{5}}{p_{2}}}{1+\frac{\gamma +1}{\gamma -1}\frac{p_{5}}{p_{2}}}\right)\times \frac{T_{2}}{T_{1}}$$

(Equation 4) $$\frac{p_{5}}{p_{1}}=\left[\frac{2\gamma M^{2}-\left(\gamma -1\right)}{\gamma +1}\right]\left[\frac{\left(3\gamma -1\right)M^{2}-2\left(\gamma -1\right)}{\left(\gamma -1\right)M^{2}+2}\right]$$

P_1_ and T_1_ are the initial pressure and temperature of the driven sections, and they are 0.06 bar and 300 K, respectively.

#### Characterization

##### Transmission electron microscope (TEM) analyses

The nanoscale morphology and structure of the shock-synthesized BCN were analyzed by transmission electron microscopy using a Titan 300 Themis D3391 (Thermo Fisher Scientific) equipped with a Schottky field-emission gun and operated at 300 kV. This instrument enables high-resolution imaging of lattice fringes and parallel acquisition of structural and chemical information via selected-area electron diffraction (SAED) and energy-dispersive X-ray spectroscopy (EDS). For sample preparation, a small amount of tBCN powder was dispersed in ethanol and sonicated for a few minutes to obtain a well-dispersed nanosheet suspension. A drop of the resulting dispersion was deposited onto a holey carbon-supported copper grid and allowed to dry completely in air before imaging.

##### X-ray photoelectron spectroscopy analyses

X-ray Photoelectron Spectroscopy (XPS) was performed on an Omicron system (Omicron Nanotechnology Ltd, Germany) equipped with an Al Kα (1,486.7 eV) X-ray source. To avoid background from conventional carbon tape, the powders were mounted on high-vacuum-compatible copper tape, which served as a low-background, conductive substrate for analysis. Core-level spectra were recorded for B 1s, C 1s, N 1s, and O 1s. Peak fitting was performed after Shirley background subtraction, with binding energies referenced to the C 1s sp^2^ peak at 284.8 eV.^51^

##### Fourier transform infrared spectroscopy analysis

Fourier Transform Infrared (FT-IR) spectra of the precursors and the shock-synthesized BCN sample were recorded on a Nicolet iS50 FTIR spectrometer (Thermo Scientific) equipped with an attenuated total reflection (ATR) accessory. Spectra were collected over the range 4000 to 400 cm^-1^ at a spectral resolution of 2 cm^-1^.

##### UV-visible and photoluminescence spectroscopy analyses

UV-Visible absorption spectra of the precursors and the tBCN nanosheets were recorded using a JASCO V-750 spectrophotometer. Photoluminescence spectra of the BCN nanosheets were recorded in the solid state using a Hitachi F-7000 fluorescence spectrophotometer with an excitation wavelength of 295 nm and 325 nm. The optical transition energy was calculated using the relation E= 1239.8/λ, where *λ* corresponds to the emission peak wavelength.^35^

##### Electrochemical measurements

Electrochemical measurements were performed using a standard three-electrode setup on a CHI 660E electrochemical workstation.^52^ The working electrode was a modified glassy carbon electrode (GCE), the counter electrode was a graphite rod, and the reference electrode was an Ag/AgCl electrode. The working electrode was fabricated by dispersing 1 mg of turbostratic boron carbon nitride (tBCN) in a water-ethanol mixture (4:1 v/v) and 80 μL of a 5 wt% Nafion binder, followed by ultrasonication for 10 minutes to obtain a homogeneous catalyst ink. Subsequently, 5 μL of the resulting ink was drop-cast onto the GCE surface (geometric area 0.07 cm^2^) and dried at room temperature prior to measurements. All electrochemical tests were performed in 1 M KOH at room temperature. Linear sweep voltammetry (LSV) was recorded at a scan rate of 2 mV s^−1^.

### Quantification and statistical analysis

Sample morphology was characterized by high-resolution transmission electron microscopy (HRTEM), and the resulting micrographs were analyzed with ImageJ software. Electrochemical data were acquired using an electrochemical workstation. All experimental graphs were generated from the original data using Origin software, while schematic illustrations and experimental diagrams were prepared in the open-source software Inkscape.

### Additional resources

This study has not generated or contributed to a new website/forum.
