# Supplementary material for: Kinetically controlled shock-wave route to turbostratic boron-carbon-nitride nanosheets
Source: iScience. 2026 Jul 30;29(8):116978. doi: 10.1016/j.isci.2026.116978 (PMC13452224; doi:10.1016/j.isci.2026.116978)
Supplement: Supplementary file 1 [file mmc1.pdf]

iScience, Volume *29*

Supplemental information

Kinetically controlled shock-wave route to turbostratic boron-carbon-nitride nanosheets

Prakash Velampatti Selvaraj and Vijayanand Chandrasekaran

1. High-resolution TEM with EDS analysis of tBCN nanosheets:

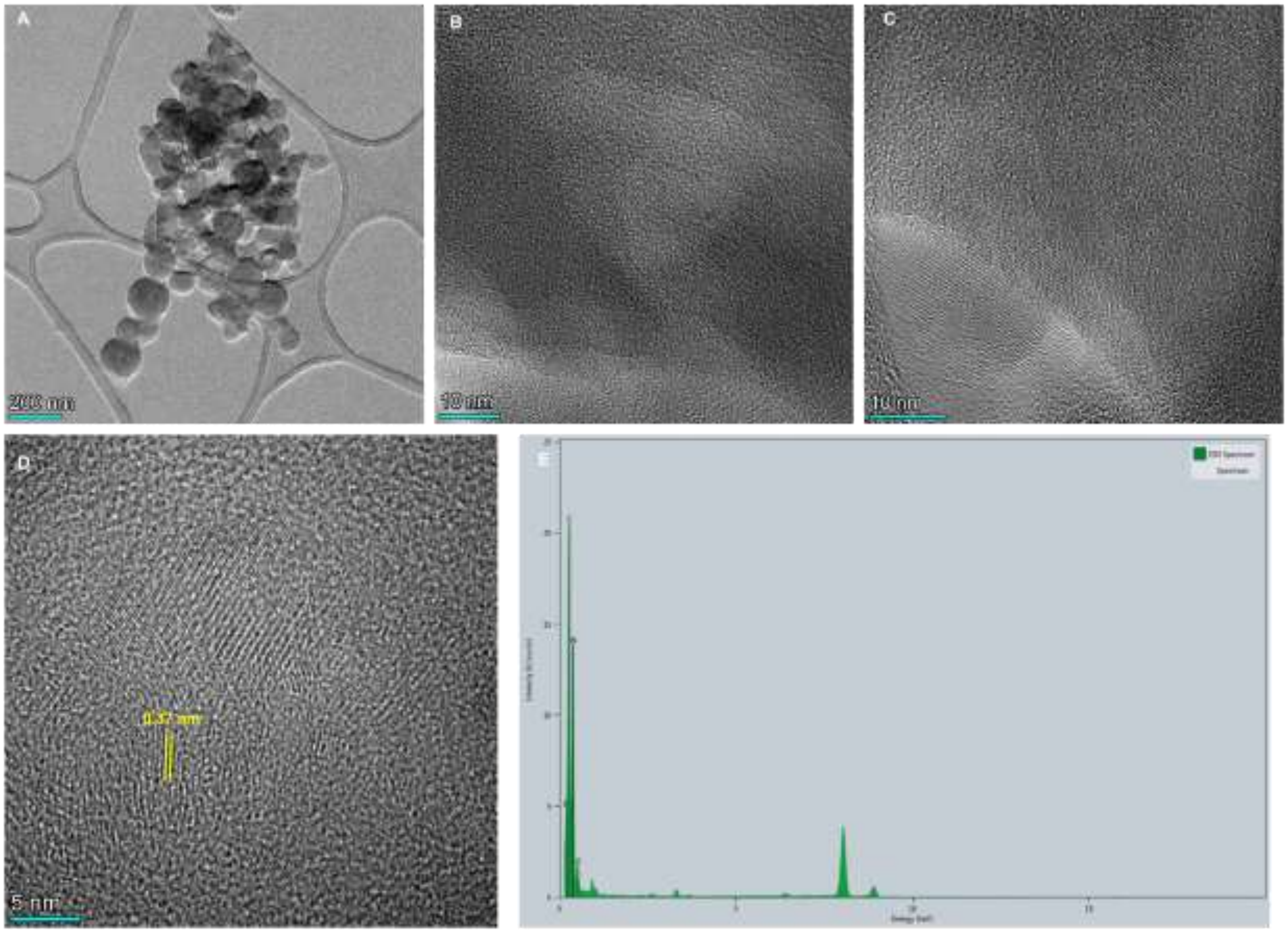

Figure S1. HRTEM and EDS analysis of shock-synthesized turbostratic BCN nanosheets. (**A**) TEM image of tBCN nanosheets showing overall morphology (scale bar, 200 nm).

(**B, C**) HRTEM images of tBCN nanosheets (scale bars, 10 nm).

(**D**) HRTEM image revealing locally ordered lattice fringes with an interlayer spacing of 0.37 nm, indicating high in-plane crystallinity within the turbostratic framework (scale bar, 5 nm).

(**E**) EDS spectrum of the shock-processed sample, confirming a ternary BCN composition with B, C, and N as the major elements and only trace oxygen.

2. XPS analyses of tBCN nanosheets:

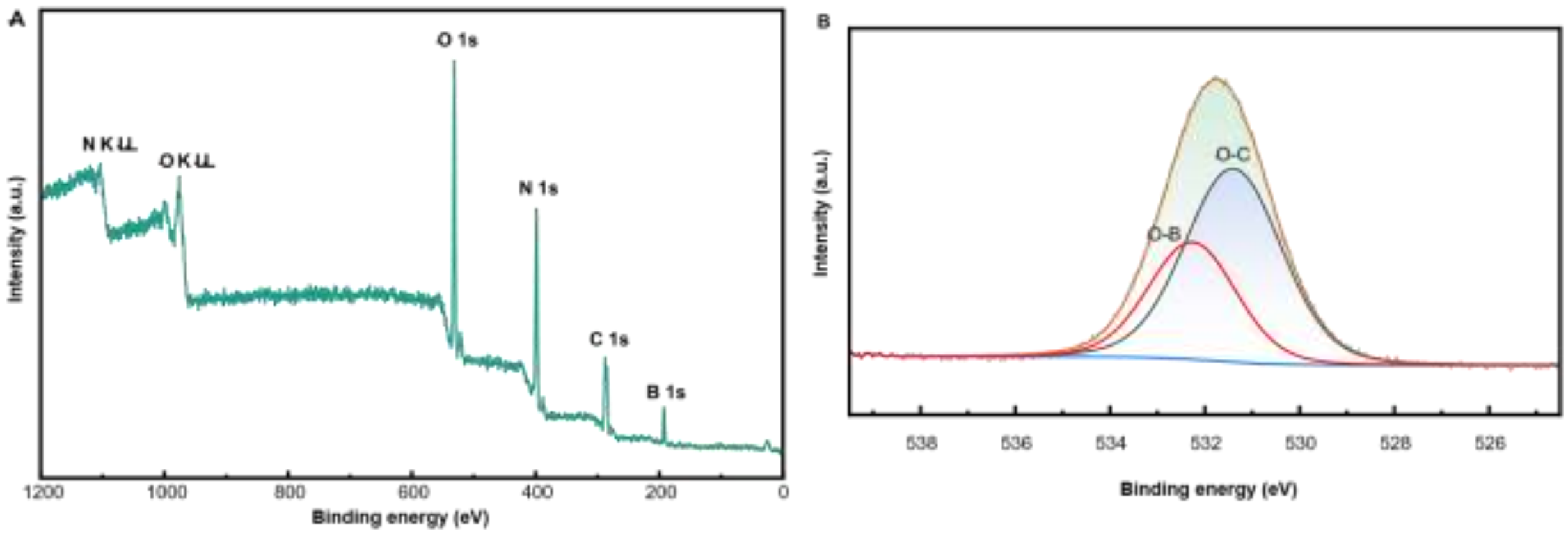

Figure S2. XPS survey and core-level spectrum of shock-synthesized tBCN nanosheets. (**A**) Wide-scan XPS spectrum showing boron, carbon, and nitrogen as the dominant elements of the tBCN nanosheets. (**B**) O 1s core-level spectrum resolved into signals associated with O-C and O-B coordination environments.

3. Band gap analysis of tBCN nanosheets:

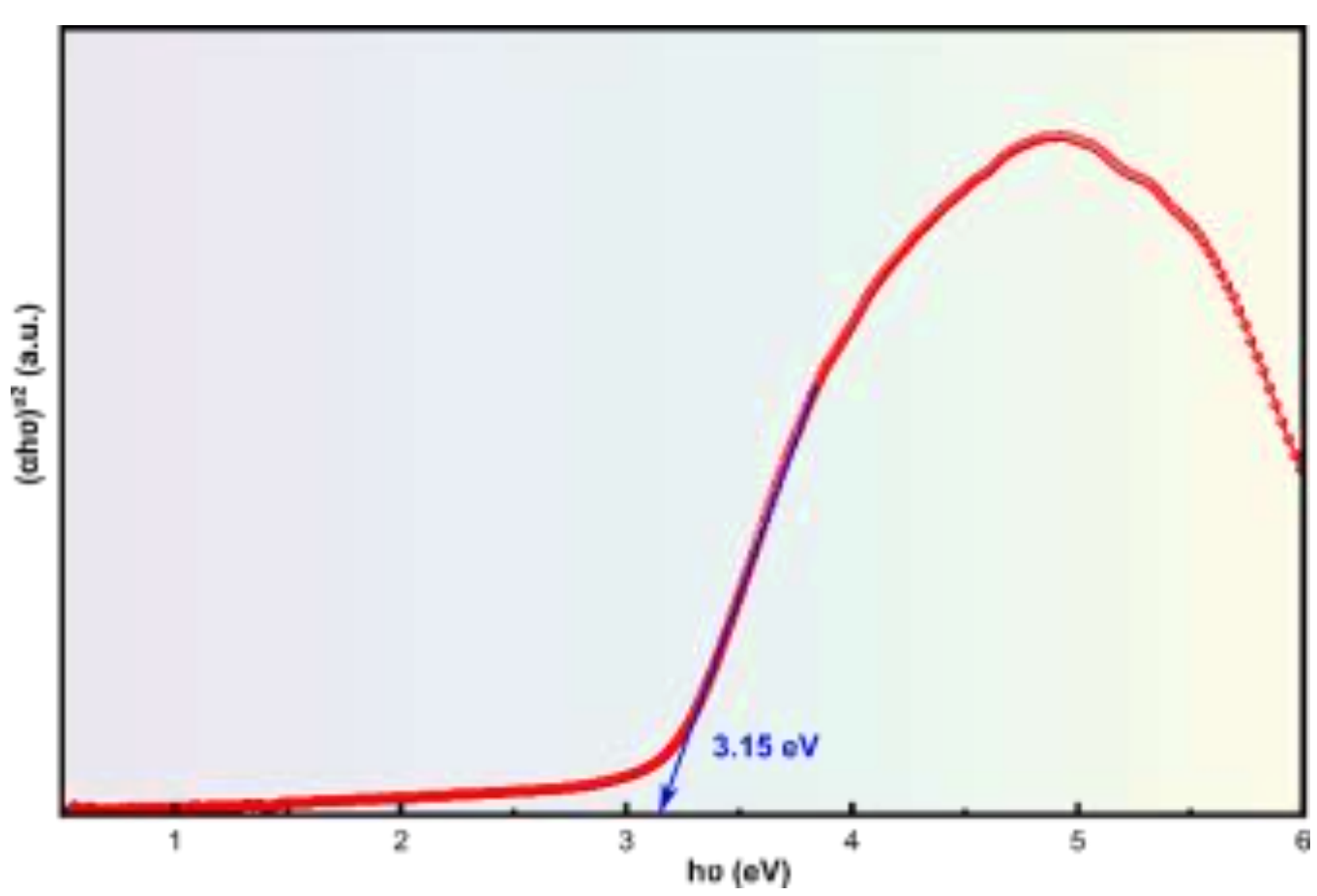

Figure S3. Optical band gap of the tBCN nanosheets derived from the absorption data using the Tauc plot method.

Table S1. EDS elemental percentages of tBCN nanosheets.

Z	Element	Family	Atomic Fraction (%)	Atomic Error (%)	Mass Fraction (%)	Mass Error (%)	Fit error (%)
5	B	K	18.48	3.62	15.92	1.8	0.02
6	C	K	47.91	13.41	45.84	10.55	1.25
7	N	K	29.05	8.13	32.42	7.45	0.9
8	O	K	4.56	1.28	5.82	1.34	0.48

METHODS S1. Shock-wave synthesis benchmarked against the twelve Green Chemistry principles.

4. Green Chemistry Principles:

From a green chemistry perspective, the shock-wave route satisfies several of the 12 principles and compares favorably with established BCN syntheses[1,2].

Reaction Mass Efficiency (RME)

For a representative run: Melamine (126 mg) + Boric acid (124 mg) → tBCN (165 mg) (no further purification), with empirical composition $BC_4N_{6.3}$ and major gaseous products H_2O, NH_3, and minor CO_2.

$$RME = m_{product} / \sum m_{reactants} \times 100\%$$

$$RME = 165 / 250 \times 100\% \approx 66\%$$

So, the reaction mass efficiency is about 66%, which is high for a solid-state route without post-synthetic purification.

Process Mass Intensity (PMI) and E-Factor (Core Synthesis)

For the core single-shock processing step (no solvents or additives).

$$PMI = \sum m_{inputs} / m_{products} = 250 / 165 = 1.52$$
$$E\text{-}Factor = PMI - 1 \approx 0.52$$

Thus, for each gram of tBCN produced, only ~0.5 g of material is not incorporated into the product (released as gaseous products or as minor solid loss during collection). In contrast, solvothermal BCN syntheses using hundreds of milliliters of DMF/ethanol per gram of product typically show PMIs in the tens to hundreds, with correspondingly large E-factors[3].

4.1. Waste prevention (P1)

The low E-factor (~0.5) and process mass intensity (PMI ~1.5) indicate that for every gram of BCN produced, only about half a gram of material leaves the process as non-product streams, with the remainder of the input mass retained in the solid. This contrasts with solvothermal and CVD-type routes, where large solvent volumes or sacrificial reagents dominate the mass balance[3–5]. In the present laboratory setup, the He or Ar driver gas leaves the shock tube as a low-pressure stream containing mainly H_2O, NH_3, and trace CO_2 generated during precursor decomposition. In the future, after each shot, this exhaust is passed through an aqueous scrubber to remove ammonia and any residual acidic species; the resulting mixture, now dominated by He (or Ar) with water vapour and minor CO_2, is then safely discharged in a fume hood. At an industrial scale, the same exhaust could instead be routed to a recovery unit, where pressure-swing adsorption or membrane modules would remove H_2O, CO_2, and NH_3, enrich the noble gas, and be followed by cryogenic distillation to deliver high-purity helium or argon suitable for reuse.

4.2. Atom economy (P2)

The empirical $BC_4N_{6.3}$ composition, together with the identification of H_2O, NH_3, and minor CO_2 as the main volatile products, indicates that boron, carbon, and nitrogen from the melamine–boric acid precursor are efficiently incorporated into the extended BCN framework, while predominantly hydrogen and a portion of the oxygen are removed as small molecules. This elemental distribution is characteristic of a high-atom-economy transformation in which the environmentally and materially significant heavy atoms (B, C, N) are retained in the desired solid, consistent with the favorable RME, PMI, and E-factor values of the solvent-free shock process.

4.3. Less hazardous synthesis (P3)

The shock-tube route relies on abundant, comparatively low-hazard solids (melamine and boric acid) rather than pyrophoric diborane, corrosive BCl_3, or halide-containing gas mixtures often used in CVD growth of BCN[6]. The dominant volatile products are water and ammonia, with only minor carbon oxides, rather than HF or HCl streams associated with the high-temperature decomposition of halide precursors.

4.4. Safer solvents and auxiliaries (P5)

The core transformation is strictly solvent-free. No organic solvent is present in the reaction zone, and any post-synthetic washing can be carried out with modest amounts of aqueous media. This avoids the liters of DMF, ethanol, or mixed organics typically required per gram of product in solvothermal BCN routes, directly reducing hazardous solvent use and associated treatment burdens[3,4].

4.5. Energy efficiency (P6)

Millisecond shock events provide the enthalpy needed to form BCN at peak temperatures of about 6000 K and elevated pressures, but only for 2-3 milliseconds. In contrast, conventional CVD or pyrolysis maintains reactors at 900–1100 °C for hours[5–7]. Although a full life-cycle energy audit is beyond the scope of this study, the combination of extremely short residence times and localized heating suggests a lower energy input per unit mass of BCN than long-duration furnace processing.

4.6. Reduce derivatives (P8)

No protecting groups, templating agents, surfactants, or conductive carbon additives are required[8]. The melamine-boric acid precursor is converted directly into turbostratic BCN in a single step, thereby eliminating additional derivatization and deprotection steps that would otherwise generate extra waste.

4.7. Catalysis (P9)

The present process operates without a catalyst yet achieves high conversion and favorable mass-based metrics. Future work could explore catalytic shock environments to further reduce peak temperature or energy demand, but the current non-catalytic configuration already performs efficiently.

4.8. Designing safer chemicals and inherently safer chemistry (P4, P12)

The turbostratic BCN product is a chemically and thermally robust 2D solid with negligible vapour pressure, thereby reducing exposure risks compared with volatile molecular reagents. As an adsorbent or electrode material, it can be immobilized in devices or packed beds and later recovered and reused, limiting environmental dispersion and reducing replacement frequency. At the process level, replacing multi-liter autoclaves, flammable organic solvents, and toxic halogenated gases with small sealed charges of low-hazard solids that decompose primarily to benign gases reduces the risk of fires, explosions, and operator exposure compared with conventional CVD lines or solvothermal reactors[3,4].

4.9. Design for degradation and real-time analysis (P10, P11)

BCN is designed primarily for durability in coatings, sorbents, and electrochemical devices, and its solid, recoverable nature facilitates recycling and reuse relative to soluble molecular products. However, detailed end-of-life and recycling studies, particularly for composites, remain underdeveloped. At present, process monitoring is *ex situ*, but the discrete, single-shot operation of the shock-tube platform is compatible with future integration of time-resolved emission or optical diagnostics, which would move the method towards real-time control and further reduce uncontrolled emissions[9,10].

REFERENCES

1. Sheldon, R.A. (2018). Metrics of Green Chemistry and Sustainability: Past, Present, and Future. ACS Sustain. Chem. Eng. *6*, 32–48. https://doi.org/10.1021/acssuschemeng.7b03505.

2. Anastas, P., and Eghbali, N. (2010). Green Chemistry: Principles and Practice. Chem. Soc. Rev. *39*, 301–312. https://doi.org/10.1039/B918763B.

3. Kaur, M., Ahmad Mir, R., Chauhan, I., Singh, K., Krishnan, U., Kumar, M., Devi, P., Pandey, O.P., and Kumar, A. (2021). Defect states induced luminescence and electrochemical studies of boron carbon nitride nanosheets. Appl. Surf. Sci. *559*, 149982. https://doi.org/10.1016/j.apsusc.2021.149982.

4. Chen, D., Huang, Y., Hu, X., Li, R., Qian, Y., and Li, D. (2018). Green Synthesis of Boron Carbonitride with High Capacitance. Materials *11*, 387. https://doi.org/10.3390/ma11030387.

5. Giusto, P., Cruz, D., Heil, T., Tarakina, N., Patrini, M., and Antonietti, M. (2021). Chemical Vapor Deposition of Highly Conjugated, Transparent Boron Carbon Nitride Thin Films. Advanced Science *8*. https://doi.org/10.1002/advs.202101602.

6. Sugino, T., Etou, Y., Tai, T., and Mori, H. (2002). Dielectric constant of boron carbon nitride films synthesized by plasma-assisted chemical-vapor deposition. Appl. Phys. Lett. *80*, 649–651. https://doi.org/10.1063/1.1436522.

7. Zhang, T., Zeng, S.F., and Wen, G. (2014). A simple precursor pyrolysis route to BCN nanoflakes. Mater. Lett. *132*, 277–280. https://doi.org/10.1016/j.matlet.2014.06.098.

8. Chen, W., Li, J.T., Ge, C., Yuan, Z., Algozeeb, W.A., Advincula, P.A., Gao, G., Chen, J., Ling, K., Choi, C.H., et al. (2022). Turbostratic Boron–Carbon–Nitrogen and Boron Nitride by Flash Joule Heating. Advanced Materials *34*. https://doi.org/10.1002/adma.202202666.

9. Biennier, L., Jayaram, V., Suas-David, N., Georges, R., Singh, M.K., Arunan, E., Kassi, S., Dartois, E., and Reddy, K.P.J. (2017). Shock-wave processing of C 60 in hydrogen. Astron. Astrophys. *599*. https://doi.org/10.1051/0004-6361/201629067.

10. Chakraborty, S., Yurchenko, S.N., Georges, R., Simon, A., Lacinbala, O., Chandrasekaran, V., Jayaram, V., Dartois, E., Kassi, S., Gusdorf, A., et al. (2024). Laboratory investigation of shock-induced dissociation of buckminsterfullerene and astrophysical insights. Astron. Astrophys. *681*. https://doi.org/10.1051/0004-6361/202347035.
